# Telemedicine and health access inequalities during the COVID-19 pandemic

**DOI:** 10.7189/jogh.12.05051

**Published:** 2022-12-03

**Authors:** Proleta Datta, Leslie Eiland, Kaeli Samson, Anthony Donovan, Alfred Jerrod Anzalone, Carrie McAdam-Marx

**Affiliations:** 1 Department of Neurology, Oregon Health and Science University, Portland, Oregon, United States of America; 2 Division of Diabetes, Endocrinology & Metabolism, University of Nebraska Medical Center, Omaha, Nebraska, United States of America; 3 Department of Biostatistics, University of Nebraska Medical Center, Omaha, Nebraska, United States of America; 4 Department of Pharmacy Practice and Science, University of Nebraska Medical Center, Omaha, Nebraska, United States of America; 5 Department of Neurological Sciences, University of Nebraska Medical Center, Omaha, Nebraska, United States of America

## Abstract

**Background:**

During the COVID-19 pandemic, health systems rapidly introduced in-home telehealth to maintain access to care. Evidence is evolving regarding telehealth’s impact on health disparities. Our objective was to evaluate associations between socioeconomic factors and rurality with access to ambulatory care and telehealth use during the COVID-19 pandemic.

**Methods:**

We conducted a retrospective study at an academic medical centre in midwestern United States. We included established and new patients who received care during a one-year COVID-19 period vs pre-COVID-19 baseline cohorts. The primary outcome was the occurrence of in-person or telehealth visits during the pandemic. Multivariable analyses identified factors associated with having a health care provider visit during the COVID-19 vs pre-COVID-19 period, as well as having at least one telehealth visit during the COVID-19 period.

**Results:**

All patient visit types were lower during the COVID-19 vs the pre-COVID-19 period. During the COVID-19 period, 125 855 of 255 742 established patients and 53 973 new patients had at least one health care provider visit, with 41.1% of established and 23.5% of new patients having at least one telehealth visit. Controlling for demographic and clinical characteristics, established patients had 30% lower odds of having any health care provider visit during COVID-19 vs pre-COVID-19 (adjusted odds ratio (aOR) = 0.71, 95% confidence interval (CI) = 0.698-0.71) period. Factors associated with lower odds of having a telehealth visit during COVID-19 period for established patients included older age, self-pay or other insurance vs commercial insurance, Black or Asian vs White race and non-English preferred languages. Female patients, patients with Medicare or Medicaid coverage, and those living in lower income zip codes were more likely to have a telehealth visit. Living in a zip code with higher average internet access was associated with telehealth use but living in a rural zip code was not. Factors affecting telehealth visit during the COVID-19 period for new patients were similar, although new patients living in more rural areas had a higher odds of telehealth use.

**Conclusion:**

Healthcare inequities existed during the COVID-19 pandemic, despite the availability of in-home telehealth. Patient-level solutions targeted at improving digital literacy, interpretive services, as well as increasing access to stable high-speed internet are needed to promote equitable health care access.

The United States (US) declared a public health emergency due to the COVID-19 pandemic on January 13, 2020. Subsequently, the Center for Medicare and Medicaid Services (CMS), followed by other insurance payers, expanded access to and increased reimbursement for telehealth services. Specifically, the 2020 Coronavirus Preparedness and Response Supplement Appropriations Act enacted in March 2020 allowed for reimbursement of home telehealth visits by video or telephone. The goals of expanding telehealth coverage were to mitigate gaps and prevent surges in demand for patient care [1,2]. Expanded coverage led to rapid telehealth implementation, allowing for in-person ambulatory visits to be replaced by telehealth visits [3-7] in efforts to decrease the transmission of the SARS-CoV-2 virus among patients and providers and conserve personal protective equipment [8].

Prior to the COVID-19 pandemic, telehealth was primarily used in special situations, particularly in rural areas and with military veterans to address health care needs in underserved populations [9,10]. In these settings, telehealth was effective and delivered similar outcomes to in-person care [11], while decreasing patient travel times [10,12-15]. Many believe that maintaining telehealth reimbursement in the post pandemic era will lead to improved health equity and outcomes by reducing geographic and other barriers to accessing care [16,17], but data supporting this hypothesis is limited.

Several studies have evaluated disparities in telehealth use during the early stages of the COVID-19 pandemic. The most consistent finding was that older, non-white patients who have Medicaid or are uninsured and who live in rural areas were less likely to have a telehealth visit compared to white, English-speaking patients with commercial insurance living in non-rural areas [7,12,18]. A study conducted in New York City used zip code mapping and found disparities in telehealth use in underserved and minority communities [19]. However, a study conducted in the Kaiser Permanente system in Southern California found that Hispanic and low-income patients had the largest increase in telehealth use following its introduction [6]. These results suggest that telehealth may not be a ubiquitous solution to health care access barriers but benefit specific populations and models of health care delivery.

We aimed to evaluate socioeconomic factors and the geospatial distribution associated with access to care and telehealth use during the COVID-19 pandemic in a large population of rural and urban patients receiving care through a US academic health system. We hypothesized that telemedicine would contribute to the efforts to maintain access to outpatient care, including in remote areas, which would have been otherwise negatively impacted by the COVID-19 pandemic. However, this access to care may be muted in patients from socioeconomic backgrounds known to face health care disparities.

## METHODS

### Study design and setting

This retrospective, cross-sectional study included patients treated by primary care and speciality providers in clinics owned and operated by the University of Nebraska Medical Center (UNMC), Omaha, Nebraska, in the United States. UNMC is a large midwestern academic tertiary health care centre in the United States with over a million outpatient visits annually. Telehealth was provided via a video platform embedded in the health system’s electronic health record (EHR). Telephone visits were used when video visits were not feasible.

### Data and study timeline

The study protocol was approved by the medical centre’s Institutional Review Board. We collected deidentified data from all in-person and telehealth ambulatory provider visits from the EHR system from March 16, 2017, through March 15, 2021. We used the one-year COVID-19 period defined as March 16, 2020, to March 15, 2021, as the study period of interest. COVID-19 period data were compared to a baseline pre-COVID-19 period from March 16, 2019, to March 15, 2020. To determine whether patients were established with a medical centre provider during these time periods, we also identified corresponding establishment timeframes which ran for two years prior to the start of each period of interest (Supplemental Figure 1 in the **Online Supplementary Document**). Patients who were seen during and were non-deceased at the end of an establishment timeframe were included in the “established” patient denominator for the subsequent period of interest.

### Study cohort

We identified “established” and “new” patient cohorts for each period of interest. Patients with at least one ambulatory visit during the respective two-year establishment period who had a provider visit during the period of interest were defined as “established” patients for that period of interest. Patients seen during the period of interest but who did not have an ambulatory visit in the two-year establishment period were classified as “new” patients. Established patients could be in one or both periods of interest, while new patients were only new in one period of interest (and would become established in the subsequent period).

### Variables

Dependent variables included the occurrence of overall ambulatory provider visits and by in-person vs telehealth (including video and phone) delivery. We created summary variables for counts of in-person and telehealth visits, after which patients were categorized as having all in-person visits or one or more telehealth visit.

Independent variables identified for each period of interest included demographic data and diagnosis of diabetes (a common comorbidity for adjustment). For established patients, we based the patient’s age on the age at the beginning of the period of interest, while we pulled other baseline characteristics (including payer type, sex, ethnicity, and race (definitions for race and ethnicity were based on those set by the Office of Management and Budget, a part of the executive branch of the United States government)) from the patient’s last visit in the respective establishment period. An exception was the diagnosis of diabetes, which was based on having a documented ICD-10 code for type 1 or type 2 diabetes at any time during the respective establishment period. For new patients, we used baseline characteristics from their first visit in the period of interest. Due to the nature of the data warehouse, patient residential zip codes were associated with a patient’s address at the time of the data extraction (May 2021). For data not available at the patient level (ie, income and internet access), we assigned patients the value associated with their zip code as a proxy, using American Community Survey 2019 five-year estimates [20].

### Statistical analyses

For univariable statistics, we used χ^2^ tests and independent samples *t* tests or Wilcoxon Rank Sum tests (when data was highly skewed) to assess the differences in categorical and continuous variables, respectively, between groups of interest when the two groups were independent (ie, the same patient did not contribute data to both groups). We provide only descriptive statistics when groups were not independent (eg, comparing established patients between periods of interest). No steps were made to impute missing data. We thus calculated percentages for all descriptive statistics using the non-missing data within each variable as the denominator.

We conducted multivariable regression analyses to identify 1) the odds of established patients having a visit during the COVID-19 period relative to the pre-COVID-19 period, controlling for patient characteristics, and 2) patient characteristics associated with having at least one telehealth visit vs all in-person visits during the COVID-19 period (with separate models for new and established patients). We used logistic regressions when patients only contributed a single observation (eg, patients within the COVID-19 period). For outcomes where a patient could contribute multiple observations (eg, an established patient seen in both periods for a model that assessed data for both periods), we utilized generalized estimating equations to account for the correlation within patient. For all models, we presented adjusted odds ratios (aOR) with 95% confidence intervals (95% CIs), which were Bonferroni adjusted based on the number of possible pairwise comparisons that could be made within each variable. *P*-values of less than 0.05 were considered to be statistically significant. Statistical analyses were performed using SAS version 9.4 (SAS Institute Inc., Cary, NC).

### Mapping

We further summarized patient-level data at the zip code level for each period of interest. Rates were calculated and the percentage of established patients who had a visit in the associated period of interest, and the percentage of patients seen during COVID-19 with a telehealth visit for established and new patients, separately. Zip codes with less than five patients in the denominator were excluded from mapping to avoid extreme percentages. We associated summary statistics for each zip code with the centroid for that zip code, and inverse distance weighting interpolation was used to generate an estimated surface of percentages across the entire state. The mapping was done using ArcGIS Pro software version 2.7.0 (Esri Inc., Redlands, California).

## RESULTS

A total of 128 598 out of 243 248 (52.9%) established patients seen and non-deceased at the end of each establishment timeframe had at least one ambulatory provider visit during the pre-COVID-19 period; 125 855 out of 255 742 (49.2%) had at least one ambulatory provider visit during the COVID-19 period. Approximately 33.6% (65 068 out of 193 666) of all patients seen in the pre-COVID-19 period were new, while only 30.0% (53 973 out of 179 828) of all patients seen in the COVID-19 period were new.

Baseline characteristics of the patient cohorts are reported in **Table 1**. Established patients were similar between time periods, with patients being on average 50 years of age, predominately female, commercially insured, non-Hispanic White, and English-speaking. The majority ( ~ 70%) in pre-COVID-19 and COVID-19 periods lived within 30 miles of the medical center and median internet access at the zip code level was high ( ~ 88% for both periods). All baseline characteristics for new patients were statistically different between the pre-COVID-19 and COVID-19 periods, except gender. Compared to new patients in the pre-COVID-19 period, new patients in the COVID period were slightly older, more of them had Medicaid coverage, were of Hispanic ethnicity, and lived within 30 miles of the medical center.

**Table 1:** Baseline characteristics of new and established patients seen in pre-COVID-19 vs COVID-19 time periods

	Established patients*		New patients		
	**Pre-COVID-19 period (n = 128 598)†**	**COVID-19 period (n = 125 855)†**	**Pre-COVID-19 period (n = 65 068)†**	**COVID-19 period (n = 53 973)†**	***P*-value‡**
**Mean age – years, (SD)**	49.3 (21.3)	50.1 (20.8)	40.4 (22.1)	41.0 (21.6)	<0.001§
**Age in categories – n (%)**					<.0001
<19	10 280 (8.0)	8783 (7.0)	11 128 (17.1)	8539 (15.8)	
19-34	25 685 (20.0)	24 421 (19.4)	18 263 (28.1)	14 976 (27.8)	
35-49	24 479 (19.0)	24 345 (19.3)	12 145 (18.7)	10 906 (20.2)	
50-64	31 758 (24.7)	31 426 (25.0)	12 053 (18.5)	10 174 (18.9)	
65-79	28 280 (22.0)	28 952 (23.0)	9001 (13.8)	7465 (13.8)	
80+	8116 (6.3)	7928 (6.3)	2478 (3.8)	1913 (3.5)	
**Sex – n (%)**					0.68
Male	52 575 (40.9)	51 169 (40.7)	29 724 (45.7)	24 718 (45.8)	
Female	76 016 (59.1)	74 680 (59.3)	35 329 (54.3)	29 236 (54.2)	
**Insurance type – n (%)**					<0.001
Commercial	60 369 (49.4)	58 676 (49.0)	33 175 (56.8)	26 187 (54.9)	
Medicaid	10 511 (8.6)	9 747 (8.2)	6159 (10.6)	5894 (12.4)	
Medicare	43 261 (35.4)	43 299 (36.2)	14 274 (24.4)	11 573 (24.3)	
Self-paid	1352 (1.1)	1172 (1.0)	859 (1.5)	553 (1.2)	
Other	6613 (5.4)	6784 (5.7)	3939 (6.7)	3507 (7.4)	
**Zip code level income – mean (SD)**║	$67 849 (22 797)	$68 116 (22 840)	$67 981 (23 245)	$67 457 (23 240)	<0.001§
**Race – n (%)**					<0.001
White	105 038 (82.3)	103 011 (82.5)	50 853 (80.8)	41 759 (80.6)	
Black	11 877 (9.3)	11 521 (9.2)	4636 (7.4)	4072 (7.9)	
Asian	2778 (2.2)	2626 (2.1)	1920 (3.1)	1260 (5.4)	
Other	7937 (6.2)	7724 (6.2)	5527 (8.8)	4754 (9.2)	
**Ethnicity – n (%)**					<0.001
Hispanic	6448 (5.1)	6 439 (5.2)	4648 (7.4)	4276 (8.2)	
Non-Hispanic	120 900 (94.9)	118 215 (94.8)	58 320 (92.6)	47 626 (91.8)	
**Preferred language – n (%)**					0.001
English	125 604 (97.8)	122 849 (97.8)	62 035 (96.5)	51 634 (96.4)	
Spanish	1865 (1.5)	1817 (1.5)	1492 (2.3)	1393 (2.6)	
Other	995 (0.8)	964 (0.8)	746 (1.2)	557 (1.0)	
**Distance in miles from medical centre – median (IQR)**	9.5 (5.0-44.3)	9.1 (5.0-37.7)	10.3 (5.5-48.6)	9.6 (5.4-47.5)	<0.001¶
**Rurality – n (%)**					<0.001
Nonurban-adjacent rural	6611 (5.2)	6328 (5.0)	4166 (6.5)	3367 (6.3)	
Urban-adjacent rural	12 392 (9.7)	11 970 (9.5)	8429 (13.1)	6819 (12.7)	
Urban more than 30 miles	17 853 (13.9)	16 166 (12.9)	9991 (15.5)	7416 (13.8)	
Urban within 30 miles	91 172 (71.2)	91 015 (72.5)	41 872 (65.0)	36 050 (67.2)	
**Zip code level internet access – mean (SD)**║	87.7 (6.8)	87.8 (6.8)	87.7 (6.9)	87.6 (6.9)	0.003§
**Death prior to COVID-19 period – n (%)**	1486 (1.2)	1474 (1.2)	415 (0.6)	331 (0.6)	0.59

SD – standard deviation, IQR – interquartile range

*164 168 total unique patients, where 90 285 (55.0%) were seen in both periods. No *P*-values for established patients, as patients may contribute to both time periods.

†Time periods: pre-COVID-19 = March 16, 2019, to March 15, 2020; COVID-19 = March 16, 2020, to March 15, 2021.

‡*P*-values for COVID vs pre-COVID-19 for new patients. χ^2^ tests used unless otherwise indicated.

§*P*-value from independent samples *t* test.

¶*P* value from Wilcoxon rank sum test.

║Based on zip code of patient residence, reporting the median of zip code – level mean (SD) values for income and internet access.

In multivariable regression analyses, established patients were less likely to have any ambulatory provider visit (in person or telehealth) in the COVID-19 period compared to the pre-COVID-19 period (aOR = 0.71, 95% CI = 0.698-0.71), controlling for demographic and clinical characteristics (Supplemental Figure 2 in the **Online Supplementary Document**).

**Figure 1:**
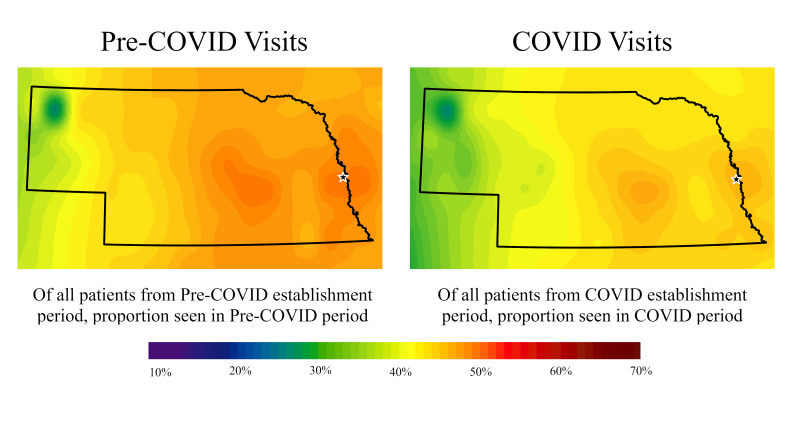
Proportion of Established patients with at least one provider visit during pre-COVID-19 and COVID-19 time periods (in-person or telehealth). White star indicates main Nebraska Medicine Campus. We created the maps based on data summarized at the zip code level. We used the centroids of zip codes for inverse distance weighting interpolation to generate estimated surfaces. For maps of percentages, we excluded zip-codes with denominators less than or equal to five to help avoid extreme percentages. Excluded zip codes are more common in the western part of the state, which can result in large areas of extreme percentages where areas with missing data are estimated by the few non-missing, extreme percentage areas around it.

**Figure 2:**
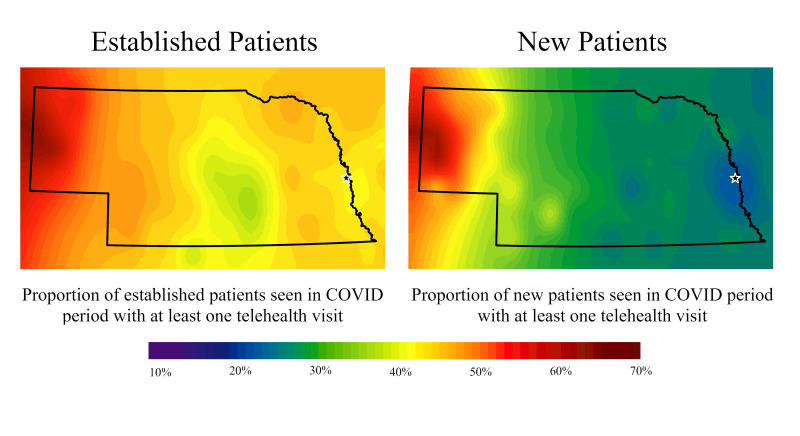
Proportion of patients with a provider visit during the COVID-19 period of which at least one visit was delivered via telehealth for established and new patients. White star indicates main Nebraska Medicine Campus. We created the maps based on data summarized at the zip code level. We used the centroids of zip codes for inverse distance weighting interpolation to generate estimated surfaces. For maps of percentages, we excluded zip-codes with denominators less than or equal to five to help avoid extreme percentages. Excluded zip codes are more common in the western part of the state, which can result in large areas of extreme percentages where areas with missing data are estimated by the few non-missing, extreme percentage areas around it.

The spatial distribution of the proportions of established patients seen in the pre-COVID-19 and COVID-19 periods, and the proportions of established and new patients having at least one telehealth visit in the COVID-19 period can be seen in **Figure 1** and **Figure 2**. These maps represent percentages of patients in an area rather than overall counts (ie, higher percentage areas do not mean there are greater number of patients in those areas). The percentage of established patients with a visit (in person or telehealth) in either period of interest is similar across the two time periods. There were higher percentages around the medical centre and other larger cities in east central Nebraska, and lower percentages in more rural and western areas of the state. Further, percentages shifted downward in the COVID-19 period, indicating that fewer established patients were seen relative to just one year prior (**Figure 1**).

The percent of established patients with at least one telehealth visit increased from 0.4% in the pre-COVID-19 period to 41.1% in the COVID-19 period. Similarly, 1% of new patients had a telehealth visit during the pre-COVID-19 period vs 23.5% of new patients during the COVID-19 period (*P* < 0.001).

Maps illustrating the percentage of patients who utilized telehealth at least once in the COVID-19 period (**Figure 2**) highlighted a higher percentage of utilization in established patients in more rural and western parts of the state than in urban areas. Overall, the percentage of telehealth usage among established patients was much higher than in new patients. For new patients, the percentage of those who used telehealth tended to increase with increasing distance from the medical center.

### Social determinants of health and telehealth visits in the COVID-19 period

**Figure 3:**
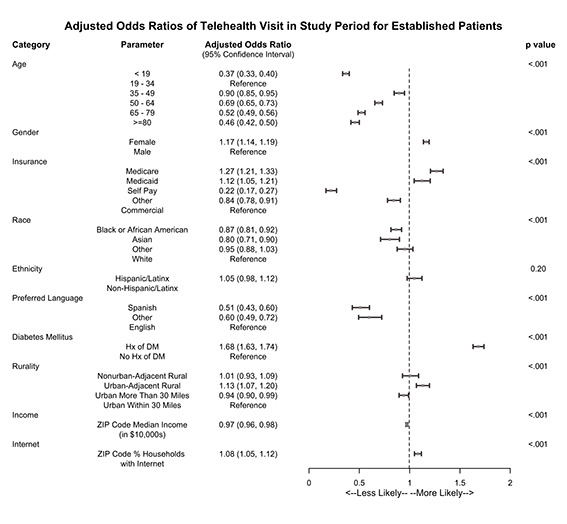
Association between social factors and the odds of having at least one telehealth visit during the COVID-19 period – established patients.

Factors associated with having at least one telehealth use during the COVID-19 period in established patients are shown in **Figure 3**. Patients younger than 19 and older than 34 years were less likely have a telehealth visit, while females were more likely to have a telehealth visit than males. Patients with Medicare and Medicaid had higher odds of having a telehealth visit compared to those with commercial insurance. Other social determinants of health associated with lower odds of having a telehealth visit include self-pay or other insurance type (relative to commercial insurance), Black or Asian race (relative to White race), non-English preferred languages, and living in a higher income zip-code. Living in a rural area was not significantly associated with telehealth use compared to residing in an urban area within 30 miles of the medical centre. However, patients living in an urban-adjacent rural zip code had slightly higher odds of having a telehealth visit, and living in an urban area more than 30 miles from the medical center was associated with lower odds of having a telehealth visit. The likelihood of having a telehealth visit was positively associated with average internet access in the patient’s zip code of residence.

**Figure 4:**
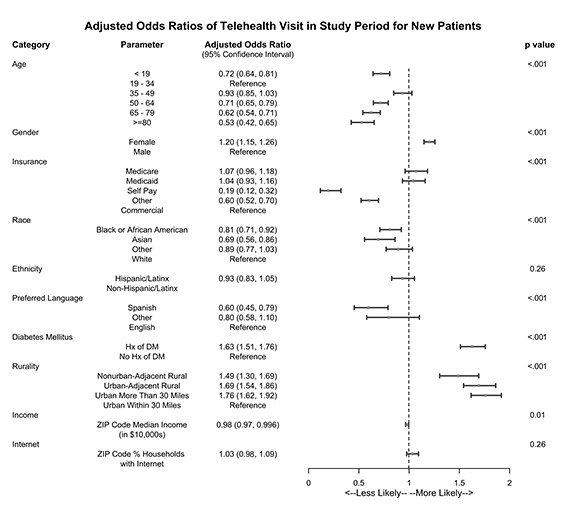
Association between clinical and social factors and the odds of having at least one telehealth visit during the COVID-19 period – new patients.

Factors associated with having at least one telehealth visit during the COVID-19 period for new patients are shown in **Figure 4**. The association between demographic characteristics and having a telehealth visit for new patients mirrored those of established patients for age, gender, race, Spanish language, and income. Self-pay patients and those with other insurance similarly had lower odds of having a telehealth visit than those with commercial insurance. However, new patients with Medicare and Medicaid were not more likely to have a telehealth visit, as was seen in established patients. Unlike the established group, new patients living in more rural areas had higher odds of telehealth use vs those living in an urban setting within 30 miles of the medical center.

## DISCUSSION

This study examined access to care and identified factors associated with ambulatory provider visits and telehealth use during the first year of the COVID-19 pandemic at a Midwestern US academic medical center. This represented a substantial shift in health care delivery due to lack of reimbursement models for in-home telehealth prior to the pandemic.

We report that, as expected, fewer established patients had a provider visit during the COVID-19 period vs the pre-COVID-19 baseline. At the same time, telehealth use increased substantially, alleviating the pandemic’s impact on access to care. Specifically, the proportion of established patients with at least one telehealth visit increased from 0.4% pre-COVID-19 to 41.2% during the COVID-19 period, although new patient use of telehealth was lower at 23.4%. Overall, we found that many patients with a telehealth visit also had at least one in-person visit during the COVID-19 period, suggesting that telehealth is an option for care delivery, but not a complete replacement for in-person visits. In-person visits remain essential per need and patient/provider preferences, dynamics that may have contributed to lower telehealth use by new patients.

The relationship between rurality, provider visits, and telehealth use provided us with useful insights. Relative to living within 30 miles of the medical center, established patients living more than 30 miles away were less likely to have a health care provider visit during COVID-19 than during the pre-COVID-19 period. However, patients living in rural areas remote from the medical center may have opted to seek care at other health care facilities not affiliated with the university. While patients from these further locales made up a smaller proportion of new patients during the COVID-19 period, they were more likely to use telehealth. A recent study reported that adults living in midwestern US and in non-metropolitan areas have decreased odds of using telehealth [21]. However, the overall relationship between telehealth use and rurality in our study was mixed and our data suggest that telehealth may have helped rural patients access care at the medical centre during COVID-19 pandemic.

Another notable finding regarding telehealth use during the COVID-19 period is that patients from zip codes with lower median incomes had higher odds of utilizing telehealth. Thus, telehealth may benefit some patients with time and/or transportation barriers to accessing care. However, patients of a minority race/ethnicity, older age, uninsured status, and/or whose preferred language was not English were less likely to use telehealth. These findings are generally consistent with the limited literature on telehealth use by social determinants of health [22,23] and suggest that work is needed to educate and develop resources to make telehealth more accessible to diverse populations.

### Strengths and limitations

The strength of this study is in its longitudinal analysis of health care provider access in a relatively stable population before and during the COVID-19 pandemic. Our inclusion criteria were broad, and our population was largely representative of the diversity in the US [24]. Furthermore, our medical centre has a large geographic and rural catchment area, and it operates primary care and specialty clinics throughout the metropolitan area. Thus, this study represents a breadth of settings and serves as a good model to study potential challenges to telehealth use.

One limitation of this study is its retrospective single center design. We did not have access to data for the patients in our cohort who may have received additional care outside of the academic medical center to help understand overall health care access. Moreover, income and internet access were not available in EHR systems and were obtained at a zip code level using ACS data that predates the COVID-19 pandemic. Data analysed by medical sub-specialty is not provided and we acknowledge that certain specialties are more amenable to telehealth than others.

In our study, telehealth included both telephone and video visits. Evaluating these visit types separately may highlight further disparities. Studies have reported that, despite interest in telemedicine, navigating technology and internet connectivity required for video visits remains a challenge in certain patient populations [25-27]. Our study was not designed to assess specific technology barriers and further investigation is warranted. Finally, we recognize that having access to internet is not equivalent to having access to broadband speeds required for telehealth visits by video conferencing. Future studies on long-term outcomes, quality metrics, effect on delays in preventative care, consumer demands, cost-effectiveness, and effects of access to COVID-19 vaccinations on telehealth use are needed.

## CONCLUSIONS

The COVID-19 pandemic has transformed health care delivery in the US by increasing access to telehealth, a model that is likely to remain in the post-pandemic world. As we move towards a health care model that includes both in-person and telehealth care, our study highlights that solely changing reimbursement for telehealth is insufficient to expand access to care. Our data reinforces that access disparities existed, despite the availability of in-home telehealth visits during the COVID-19 pandemic. Our study emphasizes the need for solutions targeting a) improving digital literacy, b) interpretive language services (including inclusion of multi-language instructions and websites), and c) access to stable high-speed internet. These findings should prompt strategies and policy changes to allow for a more equitable health care system.

## Additional material:


Online Supplementary Document
