# Supplementary material for: Telemedicine and health access inequalities during the COVID-19 pandemic
Source: J Glob Health. 2022 Dec 3;12:05051. doi: 10.7189/jogh.12.05051 (PMC9718446; doi:10.7189/jogh.12.05051)

ONLINE SUPPLEMENTARY DOCUMENT

Title: Telemedicine and health access inequalities during the COVID-19 pandemic

Authors: Proleta Datta (http://orcid.org/0000-0002-1772-8845); Leslie Eiland ; Kaeli Samson ; Anthony Donovan; Alfred Jerrod Anzalone; Carrie McAdam-Marx

Supplemental Figures

Supplemental figure 1 (Figure S1): Study Timeline

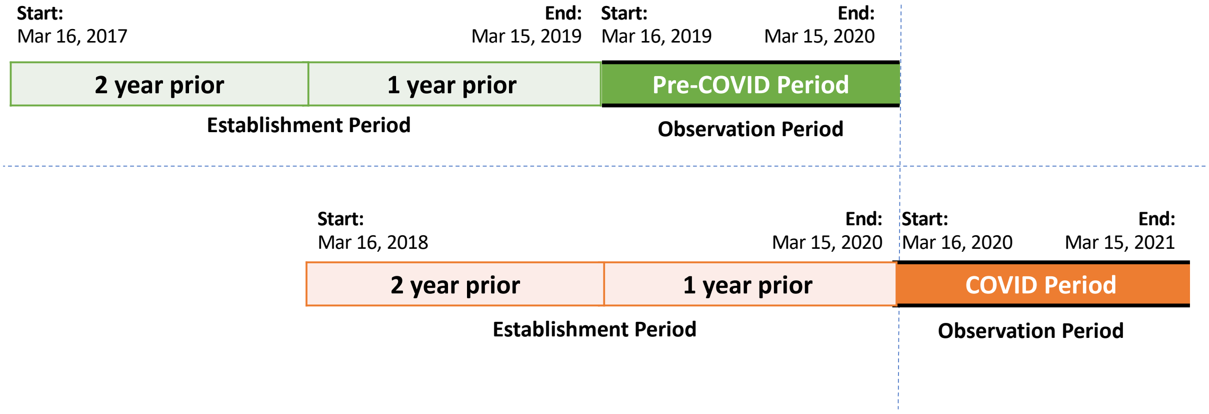

Established Patients: at least 1 outpatient visit in establishment period
New Patients: at least 1 outpatient visit in observation period but none in establishment period.

Supplemental Figure 2 (Figure S2) Adjusted Odds of Having a Provider Visit during COVID Period Relative to the Pre-COVID Period – Established Patients

Adjusted Odds Ratios of Visit in Study Period

Category	Parameter	Adjusted Odds Ratio (CI)	p value
Time Period			<.0001
	Established COVID	0.71 (0.70, 0.71)	
	Established Pre-COVID	Reference	
Age			<.0001
	<19	0.80 (0.76, 0.83)	
	19 - 34	Reference	
	35 - 49	1.39 (1.35, 1.43)	
	50 - 64	1.88 (1.82, 1.94)	
	65 - 79	1.99 (1.92, 2.07)	
	>=80	1.37 (1.30, 1.45)	
Gender			<.0001
	Female	1.21 (1.20, 1.23)	
	Male		
Insurance			<.0001
	Medicare	1.20 (1.16, 1.23)	
	Medicaid	0.98 (0.95, 1.02)	
	Self Pay	0.61 (0.56, 0.66)	
	Other	0.98 (0.94, 1.02)	
	Commercial	Reference	
Race			<.0001
	Black	1.11 (1.07, 1.15)	
	Asian	0.91 (0.86, 0.97)	
	Other	0.88 (0.84, 0.92)	
	White	Reference	
Ethnicity			0.658
	Hispanic	0.991 (0.95, 1.03)	
	Non-Hispanic		
Preferred Language			<.0001
	Spanish	0.67 (0.62, 0.73)	
	Other	0.74 (0.67, 0.82)	
	English	Reference	
Diabetes Mellitus			<.0001
	Hx of DM	1.49 (1.46, 1.52)	
	No Hx of DM	Reference	
Rurality			<.0001
	Nonurban-Adjacent Rural	0.55 (0.53, 0.58)	
	Urban-Adjacent Rural	0.55 (0.53, 0.57)	
	Urban More Than 30 Miles	0.65 (0.63, 0.66)	
	Urban Within 30 Miles		
Income			<.0001
	ZIP Code Median Income (in $10,000s)	0.99 (0.99, 0.99)	

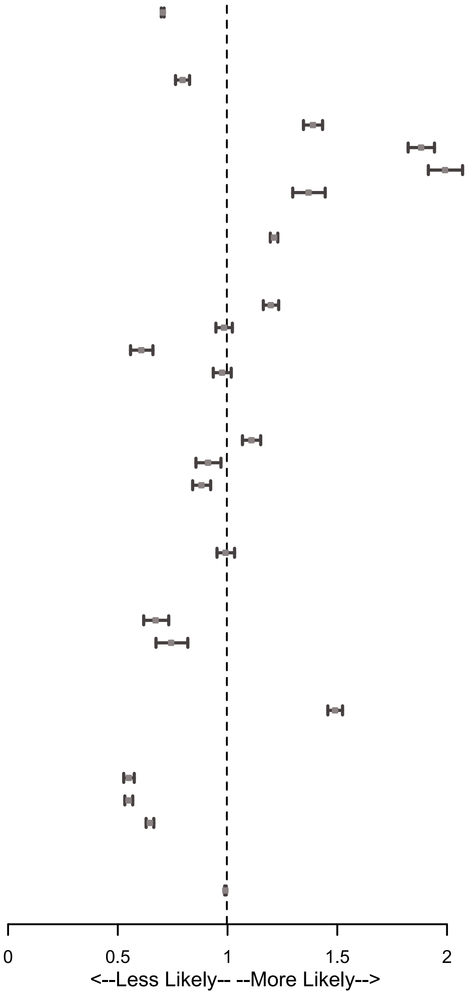

Supplement: Online Supplementary Document [file jogh-12-05051-s001.pdf]
